# High prevalence of HIV-1 transmitted drug-resistance mutations from proviral DNA massively parallel sequencing data of therapy-naïve chronically infected Brazilian blood donors

**DOI:** 10.1371/journal.pone.0185559

**Published:** 2017-09-27

**Authors:** Rodrigo Pessôa, Sabri S. Sanabani

**Affiliations:** 1 Laboratory of Dermatology and Immunodeficiencies, Department of Dermatology, Tropical Medicine Institute of São Paulo, University of São Paulo, São Paulo, Brazil; 2 Clinical Laboratory, Department of Pathology, Hospital das Clínicas, School of Medicine, University of São Paulo, São Paulo, Brazil; National and Kapodistrian University of Athens, GREECE

## Abstract

**Background:**

An improved understanding of the prevalence of low-abundance transmitted drug-resistance mutations (TDRM) in therapy-naïve HIV-1–infected patients may help determine which patients are the best candidates for therapy. In this study, we aimed to obtain a comprehensive picture of the evolving HIV-1 TDRM across the massive parallel sequences (MPS) of the viral entire proviral genome in a well-characterized Brazilian blood donor naïve to antiretroviral drugs.

**Materials and methods:**

The MPS data from 128 samples used in the analysis were sourced from Brazilian blood donors and were previously classified by less-sensitive (LS) or “detuned” enzyme immunoassay as non-recent or longstanding HIV-1 infections. The Stanford HIV Resistance Database (HIVDBv 6.2) and IAS-USA mutation lists were used to interpret the pattern of drug resistance. The minority variants with TDRM were identified using a threshold of ≥ 1.0% and ≤ 20% of the reads sequenced. The rate of TDRM in the MPS data of the proviral genome were compared with the corresponding published consensus sequences of their plasma viruses.

**Results:**

No TDRM were detected in the *integrase* or *envelope* regions. The overall prevalence of TDRM in the protease (PR) and reverse transcriptase (RT) regions of the HIV-1 *pol* gene was 44.5% (57/128), including any mutations to the nucleoside analogue reverse transcriptase inhibitors (NRTI) and non-nucleoside analogue reverse transcriptase inhibitors (NNRTI). Of the 57 subjects, 43 (75.4%) harbored a minority variant containing at least one clinically relevant TDRM. Among the 43 subjects, 33 (76.7%) had detectable minority resistant variants to NRTIs, 6 (13.9%) to NNRTIs, and 16 (37.2%) to PR inhibitors. The comparison of viral sequences in both sources, plasma and cells, would have detected 48 DNA provirus disclosed TDRM by MPS previously missed by plasma bulk analysis.

**Conclusion:**

Our findings revealed a high prevalence of TDRM found in this group, as the use of MPS drastically increased the detection of these mutations. Sequencing proviral DNA provided additional information about TDRM, which may impact treatment decisions. The overall results emphasize the importance of continuous monitoring.

## Introduction

The 2013 report from the Joint United Nations Programme on HIV/AIDS (UNAIDS) acknowledged that almost 10 million of the approximately 35 million people living with the human immunodeficiency virus type 1 (HIV-1) were receiving antiretroviral (ARV) therapy [1]. As a consequence of the introduction and advances of new ARV drugs, new infections and AIDS-related deaths have declined by 33% since 2001 and by 30% since 2005 (UNAIDS 2013). Currently, 29 ARVs in six drug classes have been approved for the treatment of HIV-1 infection, including protease inhibitors (PI), nucleoside/nucleotide reverse transcriptase inhibitors (NRTI), nonnucleoside reverse transcriptase inhibitors (NNRTI), integrase inhibitors (INI), fusion inhibitors (FI), and entry inhibitors (EI). Transmitted drug-resistance mutations (TDRM) are defined as a pre-existing resistance in individuals who have not received ARV [2]. The effects of TDRM include limited drug options and suboptimal effects of first-line ARV regimens [3], which may compromise the effectiveness of the national HIV therapeutic program by decreasing the health benefits of ARV at both the individual and population levels.

Over the past years, a handful of studies have described the prevalence of TDRM in treatment-naïve patients, which varies for different geographic regions [4–6]. Studies from the USA and Europe have demonstrated that the prevalence of TDRM in therapy-naïve subjects varies from 5 to 15% [4, 5, 7–9]. It has been shown that the TDRM in these resource-rich areas of the world have been most commonly detected to the NRTI and NNRTI, with a lower prevalence usually described for TDRM to PI [5, 9, 10]. A recent study that reviewed the current state of both acquired and transmitted drug resistance in Africa over the past 10 years (2001–2011) indicates a pooled prevalence of drug-resistance mutations of 10.6% and that Central Africa had the highest prevalence, at 54.9% [11].

The standard approach of genotypic resistance testing using bulk viral population sequencing detects only viral variants, which constitute >20% of the total viral population in a sample [12–14]. This underestimates the true overall prevalence of resistant variants, which impact significantly on the clinical management and surveillance of HIV resistance. Several studies have demonstrated that drug-resistant variants presenting at levels as low as 0.5–1% of the total viral population and that are therefore missed by bulk viral population sequencing can be of clinical importance, because they can grow rapidly under the selection pressure exerted by drugs, thus leading to therapy failure [15, 16]. The detection of HIV drug-resistance mutations that exist at very low levels in infected individuals is now technically possible through the so-called massive parallel sequencing of HIV genotyping [17–19]. Ultrasensitive genotyping can be performed by point-mutation real-time PCR assays (allele-specific PCR or AS-PCR) or with different massively parallel sequencing (MPS) platforms. The MPS technique is used to sequence short viral genomes with high redundancy and thus allow the identification and quantification of HIV-1 variants that constitute the *quasispecies* as little as 1% frequency [20, 21].

Recent findings from multiple studies reporting on the application of MPS have considerably enhanced our understanding of not only HIV biology but also viral immune escape; mother-to-child HIV transmission; and HIV epidemiology, frequency of drug-resistance variants, and viral diversity [18, 19, 22–27].

Evidence for the dissemination of drug-resistance variants in Brazil stems from reports of primary resistance in recently infected individuals (0–12.7%) as well as those with longstanding infections (5%) [28–31]. Recently, we reported the overall prevalence of 31.1% of transmitted resistance in massively parallel sequencing (MPS) data of the proviral *pol* region from treatment-naïve recently HIV-infected blood donors [22]. Here, we extended our previous study to include the analysis of the primary TDRM across the entire proviral genome using the MPS data of ARV-naïve non-recently (longstanding) infected blood donors. Besides studying the prevalence of TRDM, we also aimed to compare the profile of TDRM obtained from the PR and RT regions from plasma viruses and compare them with the MPS data from the same region of the corresponding proviruses. Previous studies have proved that sequencing the proviral DNA is an alternate source for detecting TDRM and can yield broadly similar results to those obtained in viral RNA [32]. Thus, we believe that these data will produce evidence to inform future treatment guidelines and for broader disease control efforts.

## Material and methods

### Study data

The MPS data used in the present study were derived from 134 HIV-1 near full-length genomes (NFLG) with predetermined subtype by Illumina ultra-deep sequencing technology [33]. These samples were sourced from four Brazilian states (41 from São Paulo [SP], 31 from Minas Gerais [MG], 77 from Pernambuco [PE], and 53 from Rio de Janeiro [RJ]) and were previously classified by less-sensitive (LS) or “detuned” enzyme immunoassay (Vironostika HIV-1 MicroElisa; bioMérieux, Durham, NC) or an LS chemiluminescent immunoassay (Vitros HIV-1/2 Assay; Ortho Diagnostics, Rochester, NY) as non-recent or longstanding infections being Recent Infection Testing Algorithm (RITA) reactive [22]. None of the participants had been exposed to any ARV at the time of sampling (i.e. treatment-naïve). The sequences used in this study are publicly available through the Zenodo repository browser [33, 34]; therefore, no institutional review board (IRB) approval is required.

### Patients’ characteristics

After the removal of MPS data with low coverage reads (<500x) and poor quality reads, the number of samples dropped to 128 SP (n = 18), RJ (n = 42), MG (n = 15), and PE (n = 53), and these were considered for TDRM evaluation. The average sequence length that aligned to the consensus sequence for the PR, RT, IN, and *env* is 374 bp, 1320 bp, 867 bp, and 2571 bp, respectively. The average coverage varied at each nucleotide site associated with drug-resistance mutations. Based on our recent phylogenetic analysis [33], 69.5% (89/128) of the subjects were infected with HIV-1 subtype B; other subtypes included C (1.6%; 2/128), unique BF recombinants (15.6%; 20/128), circulating recombinant forms (CRFs) (8.6%; 11/128), BC (2.3%; 3/128), CF1 (0.8%; 1/128), D (0.8%; 1/128), and CDK (0.8%; 1/128).

### Extraction of reads spanning the PR, RT, integrase (IN), and envelope (ENV) regions from HIV MPS

The compressed *fastq* files of the assembled quality controlled (99.9% accuracy of base call) paired-end reads from each viral complete genome were retrieved from the database and imported into CLC Genomics Workbench Version 9.0 (CLC Bio, www.clcbio.com). A contiguous sequence of each viral NFLG was generated and used in a second round assembly as a sample-specific reference sequence for extracting and mapping the target region of the reads. Single-nucleotide polymorphisms were identified with probabilistic variant detection modules using the default parameters in the mapping algorithm. We only considered targets with a coverage depth of ≥ 500 sequence reads per base pair in the unique regions of the genome for the analysis.

### Genotypic analyses

Evidence of transmitted HIV-1 drug resistance was defined as the presence of at least one drug-resistance mutation placed on the list of mutations published in the International Antiviral Society USA report on drug-resistance mutations (IAS-USA, 2014 update) and the World Health Organization's 2009 list of surveillance drug resistance mutations [35, 36]. The minority HIV-1 resistant variants were identified using a threshold of ≥ 1.0% and ≤ 20% of the reads sequenced. Reads with less than 1% were discarded to account for potential errors due to the error rate of PCR.

## Results

### TDRM analysis in PBMC

All samples had mutations that confer resistance to the RT or PR inhibitors (S1 Table). Among them, 23 (18%) had detectable resistance solely to NRTI, 11 (8.6%) had detectable resistance to only NNRTI, and 14 (10.9%) showed resistance to both types of inhibitors. Mutations conferring resistance to PI were found in 127 of the 128 (99.2%) investigated subjects; 108 (85%) of them had accessory mutations. Nineteen participants were found to harbor proviruses with major resistance to PI, and 15 of them had minority population variants. Thus, if we considered only the major mutations to PI, then the overall prevalence of TDRM were 44.5% (57/128), including any mutations to the NRTI and NNRTI (S2 Table). Of the 57 subjects, 43 (75.4%) harbored a minority variant containing at least one clinically relevant TDRM. Among the 43 subjects, 33 (76.7%) had detectable minority resistant variants to NRTI, 6 (13.9%) to NNRTI, and 16 (37.2%) to PR inhibitors. Four subjects (10BR_MG045, 10BR_PE014, 10BR_PE109, and 10BR_SP071) had triple-class drug resistance. Ten subjects had detectable TDRM at >20% of the viral population in the proviral DNA than in plasma. Neither major resistance-associated IN mutations (T66I/A/K, E92Q/G, T97A, Y143HCR, S147G, Q148H/R/K, and N155H) nor mutations related to fusion inhibitor (FI) resistance within the HR1 domain of the gp41 coding region (codons 36–45, crucial for ENF resistance) were detected.

### TDRM to PR inhibitors (PIs)

The analysis of TDRM revealed a total of 405 mutations associated with ARV in the PBMC in 20 codons (10, 16, 20, 30, 33, 36, 46, 60, 62, 63, 64, 69, 71, 73, 77, 82, 85, 88, 89, 93) of the entire PR region sequences, of which 20 mutations in two positions were related to major PI resistance, namely D30N (n = 2) and M46I/L (n = 18). The distribution of these mutations indicated that all participants but one (10BR_PE095) had detectable mutations in the PR (S1 Table). Of the 128 samples investigated, 19 participants had major mutations, of which 15 were derived from minority drug-resistant variant populations. The most frequent accessory mutations were M36I, V77I, and I93L, occurring in 61.1%, 41%, and 35% of the sequences analyzed, respectively. The number of mutations associated with PI resistance in each proviral protease sequence varied from zero to seven. The most abundant were sequences with two and three PI resistance-related mutations. The least frequent mutations were N88D and G73T, each present in one sample. Three samples, 10BR_SP001, 10BR_RJ083, and 10BR_SP012, each had proviral sequences with seven PI resistance-related mutations. Our analysis also revealed 61 TDRM including 18 major resistance mutations in PBMC but not in the plasma from 39 subjects. Of these 61 mutations, 31 were observed in minority drug-resistant variants, of which 16 had major resistance to PI. Fifteen of these 16 major resistances were due to amino acid substitutions at codon 46 (M46I/L), which confers high resistance to Indinavir/Ritonavir. One patient (10BR_PE058) had both D30N and M46I mutations. Similarly, the investigation of the plasma consensus sequences of the PR region yielded a total of 364 mutations associated with ARV in 20 codons (10, 16, 20, 30, 33, 36, 46, 60, 62, 63, 64, 69, 71, 77, 82, 85, 88, 89, 93), of which only two mutations at codon 30 (sample 10BR_PE014) and 46 (sample 10BR_MG045) were related to major PI resistance. Only two samples from plasma viruses (10BR_PE094 and 10BR_PE095) did not show any mutations that confer resistance to PI in the entire PR region sequences. Compared to the PBMC, our analysis of the consensus sequences of cell free viruses revealed 19 minor mutations in the plasma but not the BPMC of 15 subjects.

### TDRM to NRTIs

Forty-seven TDRM to NRTI were detected in the PBMC of 37 patients. Of these, 40 were minority variant populations with drug resistance. The analysis also revealed 42 TDRM in PBMC but not in plasma and four in plasma not detected in PBMC. F77L was the most frequently detected mutation. This accessory mutation has been reported to be associated with resistance to multiple NRTI when it occurs with Q151M [37]. Seven of the 21 subjects who had the F77L mutation also harbored drug resistance to another antiretroviral class; five of these participants had resistance to NNRTI, one had resistance to PI, one showed resistance to both PI and NNRTI, and six had multiple NRTI resistance-associated mutations. All the proviral DNA that harbored the F77L mutation were minority variant populations. The second most common NRTI mutation is the M184I, which was detected in 10 subjects. Nine of these mutations were derived from viral minority variants.

### TDRM to NNRTIs

The investigation of resistance mutations against NNRTI in the proviral DNA yielded the detection of 32 mutations from 28 patients. Six of these mutations were derived from viral minority variants. Compared to the free viruses, the analysis revealed 10 mutations in PBMC but not detected in plasma and six found in plasma but not in PBMCs. The M230I mutation, which is known to confer intermediate to high-level resistance to each of the NNRTI, was detected in six subjects and considered in this study as the most prevalent NNRTI resistance mutation. The K103N mutation associated with high-level resistance to efavirenz was detected in five subjects, and all were derived from viral populations present at frequencies more than 20%. Only one sample (10BR_RJ103) had detectable K103N in PBMC but not in plasma.

## Discussion

In this study, we determined the prevalence of TDRM from MPS data generated from 128 treatment naïve, non-recent or longstanding HIV-1 infected blood donors. Moreover, we also compared the proviral DNA sequences from patients’ PBMC to their corresponding viral RNA consensus sequences derived from published plasma viruses within the same regions to study the TDRM in both compartments. Based on the PBMC data, our results revealed that 57 of the 128 analyzed participants harbored HIV strains that were resistant to at least one class of antiretroviral agents, giving a prevalence of 44.5% of individuals with resistant viruses. This level of resistance is significantly higher than has been previously reported in treatment naïve subjects in either resource-rich or resource-poor settings, including Brazil. However, interpreting these findings as evidence of an increasing trend would be inappropriate given that the methods and samples used to generate the data in the current analysis differed from those included in previous studies; thus, comparison of the results must be done with caution. For instance, the prevalence of the TDRM observed in Latin American countries other than Brazil revealed an intermediate level in most cases, ranging between 5.7% and 8.3% [38–43]; this prevalence is significantly lower than the 44.5% rate reported in the present study. Of note, the rate estimated in this study is approximately 3–10 times higher than the rate of 5.6% to 16.3% observed amongst subjects naïve to ARV drugs within different African and high-income countries [44–50].

In Brazil, most of the studies revealed a moderate prevalence of TDRM, typically ranging from 5% to 10% [51]. Obviously, the divergence in prevalence rates between those estimated in this study and other previously published data reflects methodological differences in genotyping procedures. It is well known that Sanger sequencing has a limit of detection of approximately 20% of the total sequenced viral population [52, 53]. Thus, if we had considered this limit, we would have missed in this study the minority variants with TDRM, which represented 75.4% of the current samples. These results indicate that MPS offers improved sensitivity over Sanger sequencing for detecting resistance mutations, as has been previously demonstrated [18, 54, 55]. Other factors such as cohort characteristics as well as type of samples and infection duration may have contributed to this discrepancy. Of note, the observed rate in this group is still higher than the rate of 31.1% (14/45) of proviral TDRM detected by the same MPS technology from recently infected blood donors [22]. In a later study, Pessôa et al. [22] showed that 20% (9/45) of subjects with recent infection had detectable TDRM variants present at >20% of the viral population compared to the rate of 10% (14/128) among the longstanding infected blood donors found in this study. Also, the rate of low-abundance drug-resistant proviral HIV-1 variants was three times higher in longstanding infected subjects, at 33.7% (43/128) compared to 11.1% (4/45) in recently infected subjects. These results, however, only partially agree with the findings of Yanki et al. [8], who reported more detection of TDRM in persons with recent infection compared to those with established infection if considering only abundant drug-resistant HIV-1 variants present at >20% of the viral population. One possible explanation for this difference might be that as the time of infection goes on, purifying selection takes hold, and the increase in diversity begins to level off [56]. These results provide evidence for previous studies that suggest the presence of minority variants with drug-resistance mutations in chronically infected patients even though standard genotyping results are negative [3, 57]. A detailed analysis of the results revealed a higher frequency of TDRM for NNRTI and a lower prevalence of TDRM for NRTI if only abundant drug-resistant HIV-1 variants present at >20% of the viral population were considered. However, the opposite results were obtained when TDRM minority variants were considered in the analysis. These results are expected and probably reflect an increased use of NRTI in HIV combinational therapy.

One important observation of this study is the underestimation of the real prevalence of TDRM obtained by routine plasma genotyping that is revealed by the analysis of the MPS data of the archived viral population. The comparison of viral sequences in both sources, plasma and PBMC, would have detected 48 DNA provirus disclosed TDRM by MPS previously missed by plasma bulk analysis. Forty-two of these strains had detectable TDRM at prevalence between 1–20% of the MPS population. Other discordant data between plasma viruses and PBMC viruses in this study include the detection of 7 (12%) high-abundant drug-resistant viral variants only in the cellular compartment of the studied subject. Conversely, TDRM were only found in plasma RNA and never in PBMC-associated DNA. These results may not be surprising, since some mutations in proviral DNA may not be detected in plasma RNA or vice versa in the absence of drug pressure [58]. In fact, our data showed more detection of TDRM in the cell proviral DNA than in the plasma, as has been reported by other studies [22, 58, 59]. In this regard, we managed to detect 11 (9% of all samples) majority variants with major resistance mutations in the PR and RT genes in the PBMC of the studied subjects but not in their plasma. This result probably indicates that the MPS approach allows a comprehensive characterization of considerable diversity in the proviral DNA [23, 60].

Evidence of the transmission of strains harboring potentially resistant mutations and reduced susceptibility from therapy-naïve patients has been well documented. Altogether, these results justify the inclusion of proviral DNA as a valuable source for resistance analysis, which is in agreement with previous reports [23, 58, 59, 61]. It is known that during infection, various HIV strains are archived in the targeted cells either as wild-type or drug-resistant variants. The persistence of the archived viral DNA for a long time may potentially jeopardize the optimal efficacy of targeted drugs [62]. This is particularly important when initiating treatment in therapy-naïve subjects without available historical data or conserved samples [58].

The most commonly observed major NNRTI and PR inhibitor-associated mutations were M230I and M46I/L, respectively. F77L was the most commonly observed NRTI-associated mutation. M230L is an uncommon nonpolymorphic mutation that confers intermediate to high-level resistance to each of the NNRTI [63, 64]. This mutation is also associated with an intermediate resistance to efavirenz, which is combined in a once-daily pill with emtricitabine and tenofovir. Of note, seven individuals of the 57 participants with TDRM (12.3%) had a NNRTI mutation that would likely result in pill failure if no baseline genotyping testing was performed. This result is alarming and would likely affect the choice of empiric post-exposure or pre-exposure prophylaxis regime. Thus, our finding strongly supports the current resistance guideline that recommends baseline resistance testing before pre-exposure prophylaxis or therapy initiation.

Our study has some potential limitations. First, we should be cautious when interpreting data from studies with small sample size, aside from the inherent limitations of the cross sectional design. Second, the study focused on data from the PBMC of antiretroviral-naïve HIV-1 chronically infected blood donors, and consequently the rates of TDRM reported cannot necessarily be extrapolated to other populations of HIV-infected blood donors. Third, the lack of MPS analysis of HIV NFLG from plasma may have biased the findings from this study. Despite these limitations, the results of this analysis showed a high prevalence of TDRM among Brazilian blood donors with longstanding HIV-1 infection. These results indicate that the detection of low-frequency variants with DRM before ARV initiation can drastically increase the overall prevalence of resistance to the available ARV drugs. The high and complex TDRM patterns observed in the present study should be taken into consideration when decisions are made about initial ARV regimen for treatment-naïve patients with HIV-1. Continuous surveillance of the spread of drug-resistant HIV-1 is of utmost importance.

## Supporting information

S1 Table Drug resistance mutations detected with bulk sequencing (plasma) and deep sequencing (PBMCs)of the entire data (n = 128). (XLSX)

S2 Table Samples in which major drug resistance associated variants were detected in plasma (bulk sequencing), PBMCs (deep sequencing) or both. (XLSX)
